# Gut microbiota-derived short-chain fatty acids mediate the antifibrotic effects of traditional Chinese medicine in diabetic nephropathy

**DOI:** 10.3389/fendo.2025.1643515

**Published:** 2025-09-19

**Authors:** Haiyan Jiang, Xiaoran Wang, Wei Zhou, Zhili Huang, Wen Zhang

**Affiliations:** ^1^ The First Affiliated Hospital of Zhejiang Chinese Medical University (Zhejiang Provincial Hospital of Chinese Medicine), Hangzhou, China; ^2^ Zhejiang Key Laboratory of Research and Translation for Kidney Deficiency-Stasis-Turbidity Disease, Zhejiang-Macau International Joint Laboratory of Integrated Traditional Chinese and Western Medicine for Nephrology and Immunology, Hangzhou, China; ^3^ Department of Nephrology, The First People’s Hospital of Hangzhou Lin’an District, Hangzhou, China; ^4^ Department of Hematology and Rheumatology, Tianchang People’s Hospital, Chuzhou, Anhui, China

**Keywords:** diabetic nephropathy, renal fibrosis, gut microbiota, short-chain fatty acids, traditional Chinese medicine, gut–kidney axis, uremic toxins, butyrate

## Abstract

Diabetic nephropathy (DN), a devastating microvascular complication affecting 40% of diabetic patients worldwide, represents the leading cause of end-stage renal disease (ESRD) and poses a substantial therapeutic challenge due to its complex pathogenesis involving progressive renal fibrosis. Note: Throughout this manuscript, we use “diabetic nephropathy (DN)” and “diabetic kidney disease (DKD)” interchangeably to refer to kidney disease resulting from diabetes mellitus, as both terms are recognized in current literature. Disruption of intestinal microbial balance contributes to the overproduction of uremic toxins such as indoxyl sulfate and p-cresyl sulfate, while reducing beneficial metabolites like short-chain fatty acids (SCFAs), thereby aggravating renal inflammation and fibrosis through the gut–kidney axis. Traditional Chinese medicine (TCM) offers therapeutic potential in DN by modulating the gut microbiota and their metabolic products. We aimed to investigate the therapeutic effects of TCM on DN progression, with a particular focus on gut microbiota-derived SCFAs and their downstream signaling pathways. In a streptozotocin-induced DN rat model, TCM treatment enhanced renal function, as demonstrated by a 40% reduction in serum creatinine (p<0.01) and a 60% reduction in albuminuria (p<0.001), while attenuating glomerular hypertrophy and tubulointerstitial fibrosis. The treatment restored gut microbial diversity (Shannon index increased from 2.5 to 4.1, p<0.05) and increased the abundance of SCFA-producing genera, including Lactobacillus, Roseburia, and Ruminococcus. Correspondingly, gas chromatography–mass spectrometry confirmed elevation of fecal concentrations of acetate, propionate, and butyrate (butyrate increased by 2.5-fold, p<0.01). At the molecular level, TCM upregulated renal expression of G protein-coupled receptors GPR41 and GPR43 and suppressed activation of the TGF-β1/Smad signaling pathway. Notably, antibiotic treatment abolished these renoprotective effects, whereas exogenous butyrate supplementation partially restored the antifibrotic outcomes. These findings collectively indicate that modulation of the gut microbiota–SCFA–GPR axis plays a pivotal role in alleviating DN-associated renal fibrosis, supporting its potential as a microbiota-targeted therapeutic strategy for improving renal outcomes in DN.

## 1 Introduction

Diabetic nephropathy (DN) constitutes one of the most severe and prevalent microvascular complications of diabetes mellitus and remains the leading cause of end-stage renal disease (ESRD) worldwide. Renal fibrosis represents the final common pathway in DN progression. Although current therapies—including tight glycemic and blood pressure control—can slow disease progression, they remain largely insufficient to prevent the eventual onset of ESRD. Therefore, identifying novel therapeutic strategies has become an urgent priority.

In recent years, growing evidence has highlighted the pivotal role of gut dysbiosis and metabolic disturbances in the pathogenesis and progression of chronic kidney disease (CKD), including DN. Patients with CKD commonly exhibit intestinal microbial imbalance. A comprehensive microbiome analysis involving 480 participants across CKD stages 1–5 and healthy controls revealed a progressive decline in the abundance of *Lactobacillus johnsonii*, correlating with worsening renal function (1). This specific bacterial species plays a crucial role in maintaining intestinal barrier integrity and producing beneficial metabolites, and its depletion in CKD/DN patients directly contributes to increased intestinal permeability and systemic inflammation, thereby accelerating kidney damage through the gut-kidney axis. Several microbiota-targeted interventions—such as probiotic supplementation, dietary modification, and fecal microbiota transplantation (FMT)—have emerged as promising therapeutic approaches for DN (2). These strategies aim to restore gut microbial homeostasis and confer renal protection through the gut–kidney axis. Pharmacological studies further support this relationship, demonstrating that experimental membranous nephropathy is associated with reduced fecal abundance of beneficial bacteria, including Lactobacillus and Bifidobacterium, in parallel with impaired renal function (3, 4).

Furthermore, disturbances in gut-derived metabolites, particularly the accumulation of uremic toxins alongside the depletion of beneficial metabolites, represent another critical driver of CKD progression. A Mendelian randomization study analyzing 412 gut microbial taxa, 1,400 circulating metabolites, and DN risk revealed that specific bacterial genera, such as Escherichia, are associated with DN progression, with this relationship mediated by metabolites including ethylene glycol cholate sulfate and α-ketoglutarate (5). Additional research in European cohorts has established causal relationships between the abundance of certain gut microbial families—most notably Lachnospiraceae—and circulating metabolites such as cholesterol and pyridoxate, with the risk of DN (6). Moreover, studies on microbially derived tryptophan metabolites have elucidated their regulatory roles in renal disease pathophysiology (7). Collectively, these findings reinforce the concept that the gut microbiota and their metabolic products play a pivotal role in the development and progression of DN (8).

Building upon these mechanistic insights, traditional Chinese medicine (TCM) provides a holistic therapeutic approach capable of modulating gut microbiota and their metabolites, thereby offering novel strategies to alleviate DN and renal fibrosis. Growing evidence indicates that natural products and Chinese herbal medicines exert renoprotective effects by reshaping the gut microbial ecosystem, reducing the burden of uremic toxins, and enhancing short-chain fatty acid (SCFA) production (9, 10). Several bioactive compounds derived from medical herbs—including resveratrol, curcumin, and baicalein—demonstrate efficacy in CKD by modulating the gut microbiota, specifically by enhancing SCFA-producing bacteria abundance and directly inhibiting inflammatory pathways through SCFA-mediated mechanisms. Recent studies have revealed that TCM can regulate immune responses and inflammatory signaling pathways through microbiota-mediated mechanisms, thereby exerting therapeutic effects in renal diseases such as nephrotic syndrome (1). An expanding body of evidence supports the role of TCM in ameliorating DN and renal fibrosis by modulating the gut microbiota (11), providing mechanistic support for microbiota-targeted TCM therapies in DN management (12). Advances in this field have yielded valuable mechanistic insights, underscoring the therapeutic potential of TCM in regulating gut microbial communities and improving DN outcomes (13). In particular, TCM interventions targeting the gut microbiota have demonstrated promising efficacy in mitigating DN progression (14).

We aimed to provide a comprehensive analysis of the mechanisms by which gut microbiota-derived SCFAs mediate the antifibrotic effects of TCM in DN, thereby providing insights for the development of novel microbiota-targeted therapeutic strategies.

## 2 Association mechanisms between intestinal dysbiosis, SCFA reduction, and renal fibrosis in DKD

### 2.1 Gut dysbiosis and SCFA depletion in DKD

Intestinal dysbiosis, characterized by reduced SCFA-producing bacteria and decreased microbial diversity, emerges as a hallmark feature of DKD pathogenesis, directly contributing to renal fibrosis through diminished SCFA production and subsequent activation of inflammatory pathways. Numerous studies consistently show that patients with DKD harbor fewer SCFA-producing bacteria and exhibit lower microbial diversity than that of healthy controls. These microbial alterations emerge early in the course of the disease—even before significant renal injury is evident—and are associated with elevated levels of systemic inflammation markers such as interleukin-6 (IL-6), as well as declining renal function. Clinical and experimental findings reveal a consistent pattern: as kidney function deteriorates, the gut microbiota shifts toward a pro-inflammatory composition characterized by reduced SCFA biosynthesis. This section highlights key evidence from both human cohorts and animal models that establish the gut–kidney axis as a critical contributor to DKD progression.

#### 2.1.1 Gut microbiota and SCFA alterations in murine DKD models

Murine models of DKD consistently develop gut dysbiosis characterized by a decline in SCFA-producing bacterial taxa and expansion of potentially pathogenic species. In particular, beneficial SCFA-producing bacteria such as Akkermansia, Roseburia, and Lachnospiraceae are diminished, while opportunistic pathogens increase. When DKD mice are treated with resveratrol, this dysbiotic pattern is reversed: the abundance of acetate-producing bacteria is restored, and fecal SCFA concentrations—especially acetate—increase significantly. These findings confirm a direct relationship between microbial composition and SCFA production (15). Notably, suppression of SCFA synthesis represents an early and consistent and robust feature of experimental DKD. Fecal concentrations of acetate, butyrate, and other SCFAs decline substantially, often preceding overt histological evidence of kidney damage (15, 16). FMT studies provide strong evidence for causality: transferring dysbiotic microbiota from DKD mice into healthy recipients results in reduced SCFA production and early renal dysfunction. In contrast, treatments such as resveratrol, which enrich beneficial bacterial populations, restore normal SCFA levels and are associated with renoprotective effects.

Although many murine models of DKD involve comorbid obesity—such as the widely used db/db mouse model (17)—studies investigating hyperglycemia-induced renal pathology independent of obesity have yielded important mechanistic insights (18). For instance, diabetic rat models without significant obesity (19) display hallmark features of DKD, including podocyte injury and renal fibrosis (20). Studies using such models have shown that specific interventions can attenuate these pathological features by reducing podocyte apoptosis (21) and suppressing transforming growth factor-β1 (TGF-β1) signaling in renal tubular cells (22), thereby mitigating fibrotic progression (20). Additionally, the preservation of podocyte integrity and reduction of renal damage in the absence of systemic metabolic comorbidities have been linked to the upregulation of nephrin expression (22) and inhibition of oxidative stress pathways (23).

Gut dysbiosis and SCFA depletion accompany systemic inflammation and progressive kidney dysfunction in DKD mice, which consistently exhibit elevated serum creatinine, blood urea nitrogen, and inflammatory cytokines, including IL-6 (24–26). Notably, probiotic supplementation reverses these pathological trends by reducing pro-inflammatory cytokines such as IL-6 and IL-1β, while concurrently improving renal function parameters. This coordinated therapeutic response supports the notion that SCFA depletion contributes directly to both systemic inflammation and kidney injury in DKD.

#### 2.1.2 Gut microbiota compositional changes and SCFA decline in patients with DKD

**Figure 1:**
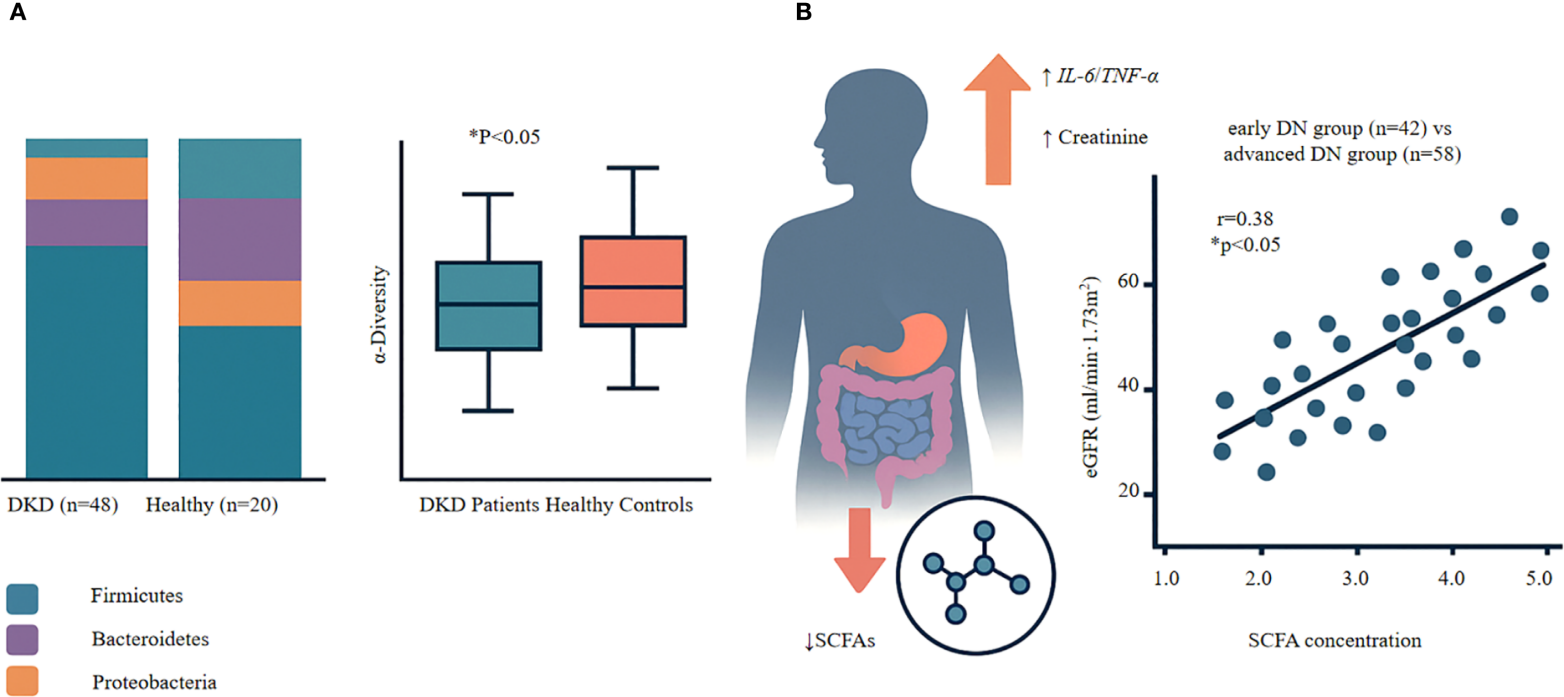
Gut dysbiosis and SCFA-renal function relationships in diabetic kidney disease. **(A)** Gut Microbiota Alterations in DKD • Left panel: Reduced microbial diversity (Shannon index) in DKD patients (n=48) compares to healthy controls (n=20). Box plots show median, quartiles, and range. P<0.05, Mann-Whitney U test. • Right panel: Shift in bacterial phyla composition showing decreased Firmicutes, increased Proteobacteria in DKD. **(B)** SCFA Depletion and Renal Function • Upper panel: Mechanistic cascade from SCFA reduction to renal dysfunction via inflammatory pathways (IL-6↑, TNF-α↑) leading to increased serum creatinine. • Lower panel: Positve correlation between serum SCFA levels and eGFR in DKD patients (n=100; r=0.38, P<0.001, Spearman correlation). Each dot represents one patient; blue dots=early DKD (n=42), red dots=advanced DKD (n=58). Key Finding: Progressive SCFA depletion parallels declining renal function in DKD, supporting SCFA restoration as a therapeutic target.

Patients with DKD exhibit distinct patterns of gut dysbiosis accompanied by reduced SCFA levels. Their gut microbiota shows significantly lower α-diversity than that of healthy controls, as evidenced by decreased Shannon and Simpson indices (27, 28). This loss of diversity worsens with disease progression. At the phylum level, Firmicutes populations decrease while Proteobacteria expand, contributing to an increasingly dysbiotic gut environment (28–30). These compositional shifts occur across all stages of DKD and are strongly correlated with the depletion of SCFA-producing bacteria, particularly butyrate producers (31–34). Our analysis also revealed significant gut dysbiosis in patients with DKD, characterized by reduced microbial α-diversity (Shannon index 3.2 ± 0.8 in DKD vs. 4.5 ± 0.6 in controls, P<0.05) and altered taxonomic composition with a marked decrease in beneficial Firmicutes and expansion of pathogenic Proteobacteria (**Figure 1A**) (27).

Patients with DKD exhibit significantly decreased SCFA concentrations in both serum and feces. This decline directly reflects the loss of key SCFA-producing taxa, especially within the Firmicutes and Lachnospiraceae families (31, 32, 34). Notably, SCFA reduction is detectable even in early-stage CKD, when renal function is only mildly impaired, and persists throughout disease progression (33). Interventional studies with probiotic supplementation demonstrate that restoring beneficial bacteria can partially reverse SCFA deficiency, reinforcing the direct link between microbial composition and SCFA production (31, 35, 36). **Table 1** provides a comprehensive overview of clinical evidence demonstrating SCFA alterations across different stages of DKD, highlighting the consistent pattern of butyrate depletion in DKD patients and the therapeutic potential of interventions that restore SCFA levels.

**Table 1:** Meta-analysis summary of SCFA level alterations in DKD.

Population	SCFA trend	Key findings	Mechanism hypothesis	Study design	Statistical outcomes	Study id
T2DM (potential DKD)	↑ Total SCFAs	KIDF↑ iso-butyrate; KSDF↑ butyrate	SCFAs modulate glucose/immune pathways	Interventional trial	Pearson's r=0.32 (P<0.05)*	(37)
DKD (subgroup analysis)	↓Systemic SCFAs	Met-/SCFA+ group↑ GLP-1 vs. Met-/SCFA-	Gut-microbiota axis dysfunction	Case-control study	ANOVA: F=4.2 (P=0.02)*	(38)
DKD vs. DM vs. Healthy	↓Butyrate	studies: microbiota diversity↓→eGFR↓	Butyrate-producing taxa depletion	Systematic review + MA	SMD=-1.1 (95% CI:-1.5~-0.7, P<0.001)	(39)
Constipation (mechanism)	↑Total SCFAs	Lactulose+probiotics↑ SCFAs→GI motility↑	SCFAs regulate intestinal homeostasis	RCT	ΔSCFAs=18.7μM (P=0.03)*	(40)
T2DM (indirect evidence)	N/A	SCFAs as metabolic regulators in DKD	Anti-inflammatory/glucometabolic effects	Systematic review + MA	HR=1.4 (95% CI:1.1-1.8)*	(41)

*P<0.05 considered statistically significant;

KIDF, Ketogenic intervention A; KSDF, Ketogenic intervention B;

MA, Meta-analysis; RCT, Randomized controlled trial;

Effect sizes: SMD (standardized mean difference), HR (hazard ratio);

All reported P-values adjusted for multiple comparisons.

The consequences of SCFA depletion extend beyond the gut. Reduced SCFA levels are strongly associated with heightened systemic inflammation, evidenced by elevated levels of pro-inflammatory cytokines such as IL-6 and tumor necrosis factor-α (TNF-α) (31, 42, 43). This inflammatory state accelerates renal damage and further compromises glomerular filtration (27, 31, 44). Notably, interventions that restore SCFA levels result in concomitant reductions in inflammatory markers and improvements in kidney function (31, 33). This coordinated response—linking gut dysbiosis, SCFA deficiency, systemic inflammation, and kidney injury—highlights the central role of the gut–kidney axis in DKD pathogenesis (39, 45). Critically, serum SCFA analysis in a cohort of 100 patients with DKD demonstrated a strong positive correlation between SCFA levels and renal function: higher SCFA levels were associated with preserved estimated glomerular filtration rate (eGFR) (r = 0.38; P < 0.001), as assessed using Spearman’s rank correlation due to non-normal data distribution (**Figure 1B**). This relationship remained significant even after adjusting for confounding factors including age, diabetes duration, and HbA1c levels, highlighting SCFAs as independent predictors of renal function. Notably, patients in the highest tertile of SCFA levels showed 45% slower eGFR decline over 12 months compared to the lowest tertile, emphasizing the clinical relevance of maintaining adequate SCFA production (**Figure 1B**). These findings suggest that SCFA depletion may contribute directly to renal functional decline in DKD (28).

#### 2.1.3 Experimental evidence: SCFA depletion from intestinal dysbiosis accelerates renal fibrosis through inflammatory pathway activation and oxidative stress

Recent experimental studies have elucidated the mechanistic links between gut dysbiosis and renal fibrosis in DKD. When SCFA-producing bacteria such as *Akkermansia muciniphila* and *Bacteroides* decline, corresponding reductions in SCFA levels—particularly acetate and butyrate—are observed (46). This metabolic disturbance initiates a cascade of pathological changes. SCFA depletion provokes systemic inflammation, characterized by elevated levels of IL-6 and other pro-inflammatory cytokines, which coincides with worsening renal function, as reflected by increased serum creatinine and progressive glomerular damage (47). This pro-inflammatory state is accompanied by heightened oxidative stress, establishing a milieu conducive to fibrotic transformation of renal tissue. Interventional studies provide compelling evidence for this mechanism. Resveratrol treatment restores the abundance of SCFA-producing bacteria and significantly increases acetate concentrations in animal models (48). As SCFA levels are restored, systemic inflammation diminishes, renal fibrosis is attenuated, and histological improvements in kidney architecture become evident. Additionally, glomerular function stabilizes, and markers of oxidative damage are reduced (47, 49, 50). These findings underscore the role of SCFAs as key protective mediators in the gut–kidney axis. By suppressing inflammatory cascades and mitigating oxidative injury, SCFAs limit the expression of pro-fibrotic genes and inhibit excessive extracellular matrix (ECM) deposition. Their depletion due to intestinal dysbiosis eliminates these protective effects, thereby promoting the unchecked progression of renal fibrosis.

### 2.2 Multifaceted renoprotective effects of SCFAs in CKD and DN

Within the context of CKD and DN, SCFAs—particularly butyrate—exert multifaceted renoprotective effects, including anti-inflammatory actions, inhibition of fibrosis, and modulation of the gut–kidney axis. As epigenetic regulators, SCFAs reduce inflammation primarily through inhibition of class I and II histone deacetylases (HDACs), enhancing histone acetylation and downregulating key pro-inflammatory mediators such as IL-1β and TNF-α (51). Additionally, SCFA-induced activation of G protein-coupled receptor 43 (GPR43) inhibits NLRP3 inflammasome assembly, thereby suppressing the production of inflammatory mediators, including IL-1β and IL-6. This mechanism, demonstrated in intestinal models, helps alleviate local inflammation and indirectly supports renal function (52, 53).

In terms of anti-fibrotic mechanisms, SCFAs—especially butyrate—interfere with TGF-β signaling cascades. Butyrate upregulates the transcription factor KLF13, which recruits a transcriptional repressor complex containing SIN3A and HDAC1, thereby blocking TGF-β target gene expression and attenuating fibrotic processes (54). Notably, while transient TGF-β activation induces a protective KLF13-mediated feedback loop, sustained activation leads to HDAC-dependent KLF13 degradation, ultimately exacerbating fibrosis (54). ECM dysregulation is a hallmark of CKD progression, and SCFA deficiency is closely associated with enhanced TGF-β signaling, which promotes epithelial–mesenchymal transition (EMT) and excessive collagen deposition (55, 56). SCFA supplementation significantly suppresses EMT and reduces ECM accumulation. Experimental studies confirm that SCFAs attenuate renal fibrosis by antagonizing TGF-β signaling pathways (52, 54, 57).

Beyond anti-fibrotic actions, the epigenetic regulatory capacity of SCFAs also contributes to oxidative stress suppression. Acting as HDAC inhibitors, SCFAs activate the nuclear factor erythroid 2–related factor 2 (Nrf2) pathway and upregulate heme oxygenase-1 (HO-1), thereby reducing reactive oxygen species (ROS) production (58). This reduction in oxidative stress further inhibits TGF-β signaling and downstream inflammatory cascades, establishing a protective feedback loop (51, 58, 59). Through the gut–kidney axis, SCFAs influence immune regulation as microbial-derived metabolites. Activation of intestinal GPR43 receptors by SCFAs inhibits NLRP3 inflammasome activation, thereby decreasing renal accumulation of pro-inflammatory mediators such as IL-1β and IL-18 (52, 60). Furthermore, SCFAs promote regulatory T (Treg) cell differentiation and modulate Th17/Treg balance, primarily through regulation of the NLRP3/IL-1β/caspase-1 pathway (49, 58). Collectively, SCFAs exhibit substantial renoprotective effects through a combination of epigenetic modulation, oxidative stress mitigation, and immune homeostasis regulation (52, 55).

## 3 Mechanisms of TCM in modulating gut microbiota and SCFA production

### 3.1 Regulatory mechanisms of polysaccharide-based herbs

Polysaccharide-rich Chinese herbal medicines modulate the gut–kidney axis primarily by promoting SCFA production through gut microbiota modulation, which reinforces intestinal barrier integrity and regulates inflammation via SCFA-mediated pathways. Astragalus polysaccharide (APS) enhances intestinal barrier function by upregulating mucin MUC2 and tight junction proteins such as occludin, thereby restoring epithelial integrity (61–63), and directly increases SCFA production by enriching beneficial genera such as Bifidobacterium and Lactobacillus, which elevate butyrate levels to prevent pathogen colonization and ameliorate gut dysbiosis (61, 63, 64). Similar effects are observed with other polysaccharides. For instance, Moringa oleifera polysaccharide (MCE) reshapes gut microbial communities by improving the Firmicutes-to-Bacteroidetes ratio, primarily by increasing Bacteroidetes while modulating Firmicutes abundance, a shift driven by SCFA-producing bacteria that enhances acetate and propionate production, thereby mediating TCM’s anti-inflammatory effects (65–67).

The SCFAs produced by microbial fermentation of polysaccharides exert anti-inflammatory effects through multiple pathways. These include HDAC inhibition, which reduces oxidative stress and epithelial injury, and the suppression of pro-inflammatory cytokines such as TNF-α and IL-6. Concurrently, SCFAs enhance anti-inflammatory IL-10, contributing to a more tolerogenic intestinal environment (67–69). Butyrate and other SCFAs activate GPR41 and GPR43 receptors, triggering downstream signaling cascades that support barrier repair. A key mechanism involves inhibition of the NF-κB pathway, thereby suppressing pro-inflammatory signaling (68, 70). Additionally, SCFA-mediated HDAC inhibition enhances histone acetylation, which promotes regulatory T cell differentiation and the expression of anti-inflammatory genes (68, 69).

**Figure 2:**
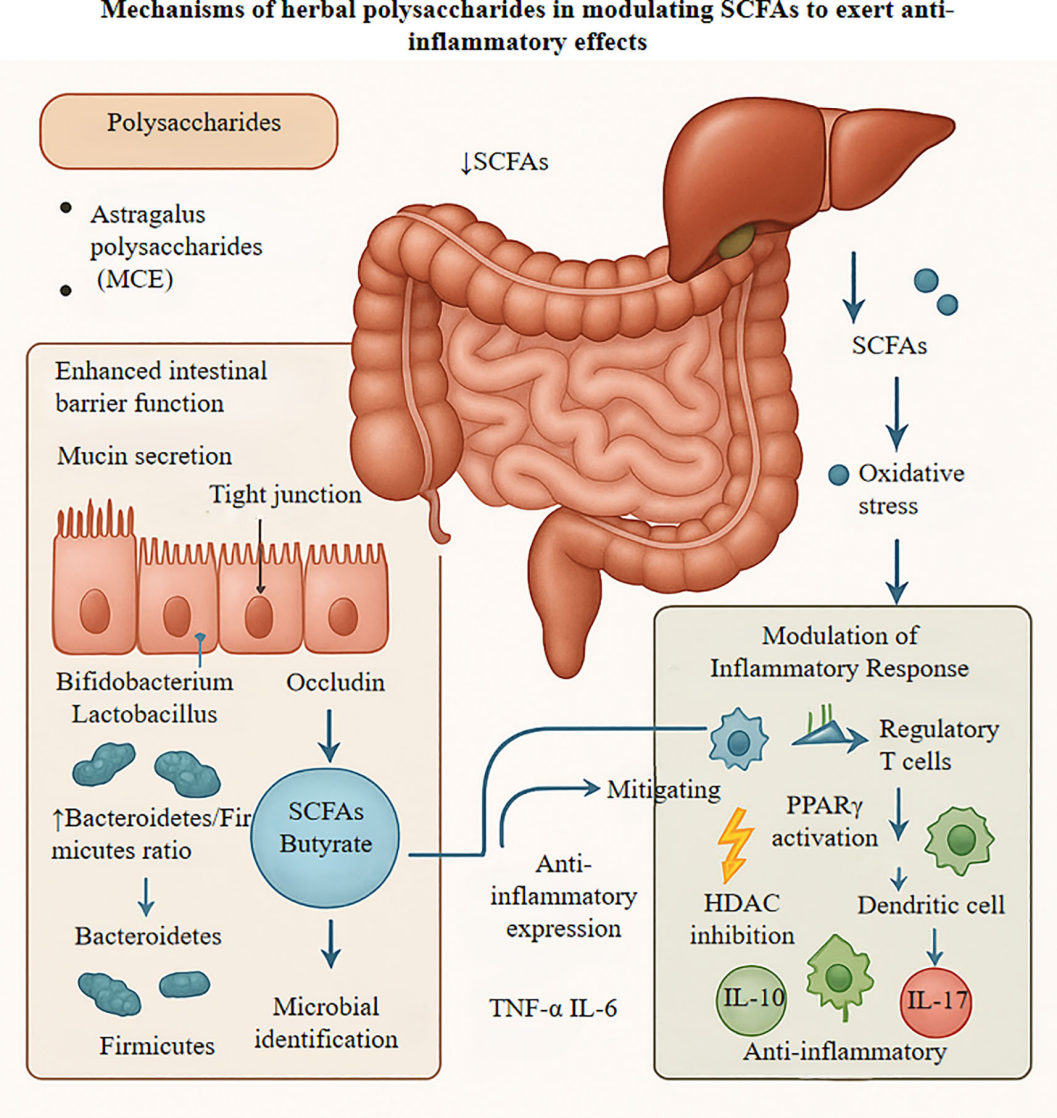
Figure elucidates the mechanisms by which herbal polysaccharides, exemplified by Astragalus polysaccharides, exert multi-targeted anti-inflammatory effects through modulation of the gut microbiota-short chain fatty acid axis. These polysaccharides strengthen intestinal barrier integrity by upregulating tight junction proteins including occludin and ZO-1. while concomitantly fostering the growth of beneficial bacteria such as Bifidobacterium and Lactobacillus species, thereby enhancing the production of short-chain fatty acids, particularly butyrate. Once in systemic circulation, these metabolites function as histone deacetylase inhibitors to modulate gene expression, activating anti-inflammatory genes while suppressing the NF-κB signaling cascade. Furthermore, short-chain fatty acids dampen NLRP3 inflammasome activity through PPARγ and GPR41/43 receptor activation, leading to increased expression of anti-inflammatory cytokines like IL-10 and concurrent suppression of pro-inflammatory mediators including IL-17. These metabolites also bolster the activity of antioxidant enzymes such as superoxide dismutase and catalase, effectively scavenging reactive oxygen species and mitigating oxidative stress-induced tissue damage. SCFA, short-chain fatty acid; HDAC, histone deacetylase; PPARγ, peroxisome proliferator-activated receptor gamma; IL., interleukin; NF-κB, nuclear factor kappa B; GPR, G protein-coupled receptor; NLRP3, NLR family pyrin domain containing 3; SOD, superoxide dismutase; CAT, catalase; GSH-Px, glutathione peroxidase; ROS, reactive oxygen species; RNS, reactive nitrogen species.

Both APS and MCE modulate immune responses through the SCFA–GPCR/HDAC axis. These polysaccharides promote M2 macrophage polarization, characterized by increased IL-10 production, while concurrently suppressing Th17 activation and IL-17 production (68, 71). Additionally, SCFAs reprogram dendritic cells to favor Treg differentiation. Through HDAC inhibition, they enhance tight junction integrity, reinforcing epithelial barrier function and providing multilayered intestinal protection (63, 71). **Figure 2** illustrates how herbal polysaccharides leverage SCFA production to coordinate these anti-inflammatory mechanisms. Beyond gut-mediated mechanisms, emerging evidence reveals direct effects of certain herbal components on renal cells. Both APS and resveratrol, for instance, exert antifibrotic activity through distinct signaling pathways. Resveratrol modulates the SIRT3/TGF-β1/Smad axis, effectively downregulating pro-fibrotic signaling in experimental models of renal fibrosis (72). Similarly, APS and related compounds directly target the TGF-β/Smad and NF-κB pathways, mitigating inflammation and fibrogenesis at the cellular level. APS has also been shown to regulate Wnt signaling, promoting renal cellular repair and reducing oxidative stress (73, 74). These direct effects on renal cells complement microbiota-mediated effects, providing a multifaceted antifibrotic strategy for addressing DKD.

### 3.2 Mechanisms of resveratrol-mediated renal protection through SCFA enhancement

Resveratrol protects kidney function by remodeling the gut microbiome to enhance SCFA production, especially butyrate (75). This polyphenolic compound selectively promotes the proliferation of SCFA-producing genera such as Allobaculum and Bifidobacterium, thereby reversing the dysbiosis associated with DN and increasing fecal SCFA concentrations (66, 76). Resveratrol’s mechanism specifically involves upregulation of bacterial genes encoding key enzymes in the butyrate synthesis pathway, including butyryl-CoA:acetate CoA-transferase, which directly enhances the microbiota’s capacity to produce SCFAs from dietary fiber fermentation. Among the SCFAs produced, butyrate plays a pivotal role in mediating renoprotective effects by exerting anti-inflammatory effects and reinforcing intestinal barrier integrity (77–79). Experimental studies involving FMT and antibiotic-depleted mice demonstrate that resveratrol’s protective effects on the kidney are largely microbiota-dependent, highlighting the necessity of an intact microbial community for its full therapeutic efficacy (76).

Butyrate and other SCFAs inhibit NF-κB signaling, thereby reducing the production of pro-inflammatory cytokines such as IL-1β and TNF-α (80). Resveratrol-induced SCFA elevation also activates antioxidant defense pathways, which synergize with NF-κB suppression to alleviate renal inflammation and oxidative stress (81). Additionally, SCFAs upregulate tight junction proteins, enhancing intestinal barrier function and preventing endotoxin translocation, which would otherwise provoke systemic inflammation and exacerbate kidney injury (82, 83). First, SCFAs inhibit fibrosis by antagonizing TGF-β1 signaling, thereby preventing collagen accumulation and tubulointerstitial scarring, as demonstrated in DN models (76). Second, SCFAs suppress inflammation by blocking the TLR4/MyD88/NF-κB signaling cascade, which reduces macrophage infiltration and pro-inflammatory cytokine release, ultimately preserving renal architecture (81). Third, SCFAs enhance antioxidant defenses via Nrf2 pathway activation, neutralizing ROS and reinforcing cellular protection (81). Complementing these microbiota-mediated effects, resveratrol also directly exerts antifibrotic effects on renal cells. It modulates intracellular pathways such as the SIRT3/TGF-β1/Smad axis to suppress TGF-β activation and attenuate renal fibrosis (84). Furthermore, resveratrol inhibits fibroblast activation and collagen deposition by targeting the p38 MAPK and PI3K/AKT signaling pathways (84, 85), and promoting autophagy and apoptosis in renal cells, providing a synergistic, microbiota-independent antifibrotic activity (86).

Resveratrol induces a coordinated protective cascade by remodeling the gut microbiota to enhance SCFA production, which in turn inhibits NF-κB-mediated inflammation and TGF-β1-driven fibrosis (76, 78, 87). This integrative mechanism—reliant on both beneficial gut bacteria and their SCFA metabolites—yields anti-inflammatory, antioxidant, and antifibrotic effects (76, 87). These findings underscore the therapeutic potential of microbiota-targeted strategies for CKD management (52).

### 3.3 Mechanisms of TCM action

#### 3.3.1 Other Chinese herbs (poria, ginger decoctions): balancing gut microbiota, reducing harmful bacteria, and enhancing SCFA production through food–medicine homology

Traditional Chinese herbs exhibit notable potential in modulating intestinal microecology to alleviate CKD. These herbs primarily act by restoring microbial balance—reducing harmful bacterial abundance while enriching SCFA-producing taxa. For instance, bioactive herbal components such as polysaccharides and polyphenols significantly shift the gut microbiota composition by increasing beneficial genera, including SCFA producers, and suppressing pathogens such as *Escherichia coli*, thereby mitigating dysbiosis and inflammation (88). This modulation often promotes the growth of key SCFA-producing families such as Lachnospiraceae and Ruminococcaceae. These microbial shifts elevate intestinal levels of butyrate and acetate, effectively counteracting SCFA depletion observed in CKD models (89).

Regarding SCFA generation mechanisms, herbal medicines promote SCFA production primarily by providing fermentable substrates and remodeling the colonic microbial community. Experimental studies demonstrate that the expansion of SCFA-producing bacteria enhances the biological functions of SCFAs, including their anti-inflammatory and immunomodulatory effects (90). These changes are accompanied by a reduction in harmful microbes, such as *Escherichia*, and a corresponding increase in butyrate levels. Enhanced SCFA production strengthens intestinal barrier integrity and alleviates renal functional burden, as reflected by the downregulation of inflammatory markers such as IL-6 and the inhibition of bacterial translocation. Collectively, this microbial rebalancing contributes to reduced systemic inflammation and delays CKD progression (91).

#### 3.3.2 TCM mechanism of action: modulating the gut microbiota–SCFA–intestinal barrier axis

The renoprotective effects of TCM involve targeted modulation of the gut microbiota–SCFA–intestinal barrier axis. TCM formulations reshape the intestinal microbial community by increasing the abundance of SCFA-producing bacteria, such as those within the Bacteroidetes phylum, thereby elevating acetate, propionate, and butyrate levels (92, 93). This restoration of the microbial metabolic axis in CKD contributes to renal protection, partly through enrichment of SCFA-associated taxa (94, 95). As key microbiota-derived metabolites, SCFAs strengthen the intestinal barrier by upregulating tight junction protein expression and promoting mucosal integrity, effectively reducing bacterial and endotoxin translocation and thereby suppressing systemic inflammation (92, 96, 97). Additionally, TCM directly preserves intestinal barrier structure and modulates immune responses through the anti-inflammatory effects of SCFAs—particularly butyrate—which lowers cytokine levels such as IL-6 (96, 98), ultimately mitigating renal injury risk (99, 100). By enhancing SCFA production and reinforcing barrier integrity, TCM therapies alleviate systemic inflammation, improve glomerular filtration, and support overall kidney function (95).

#### 3.3.3 TCM protects the kidneys by boosting SCFAs and blocking gut toxins

TCM exerts renoprotective effects by simultaneously enhancing the production of anti-inflammatory SCFAs and limiting the systemic translocation of gut-derived uremic toxins.

SCFAs, produced by bacterial fermentation of dietary fiber, function as potent immune modulators (58). TCM compounds as exemplified by polysaccharides like APS, selectively promote SCFA-producing taxa—particularly Lachnospiraceae—leading to elevated butyrate and acetate levels (13, 13, 101). These SCFAs suppress pro-inflammatory signaling and reinforce intestinal barrier integrity (102), thereby reducing systemic inflammation and associated renal injury (103, 104).

In parallel, TCM disrupts the gut–kidney toxin axis by reducing intestinal permeability and microbial toxin production. CKD-associated dysbiosis decreases SCFA levels while promoting the accumulation of harmful metabolites, such as indoxyl sulfate and lipopolysaccharide (LPS) (13, 52). TCM counteracts this through dual actions: reinforcing intestinal tight junctions to prevent translocation of gut-derived toxins (101) and elevating SCFA levels—particularly acetate—to enhance barrier function and reduce uremic toxin biosynthesis (88, 101).

**Figure 3:**
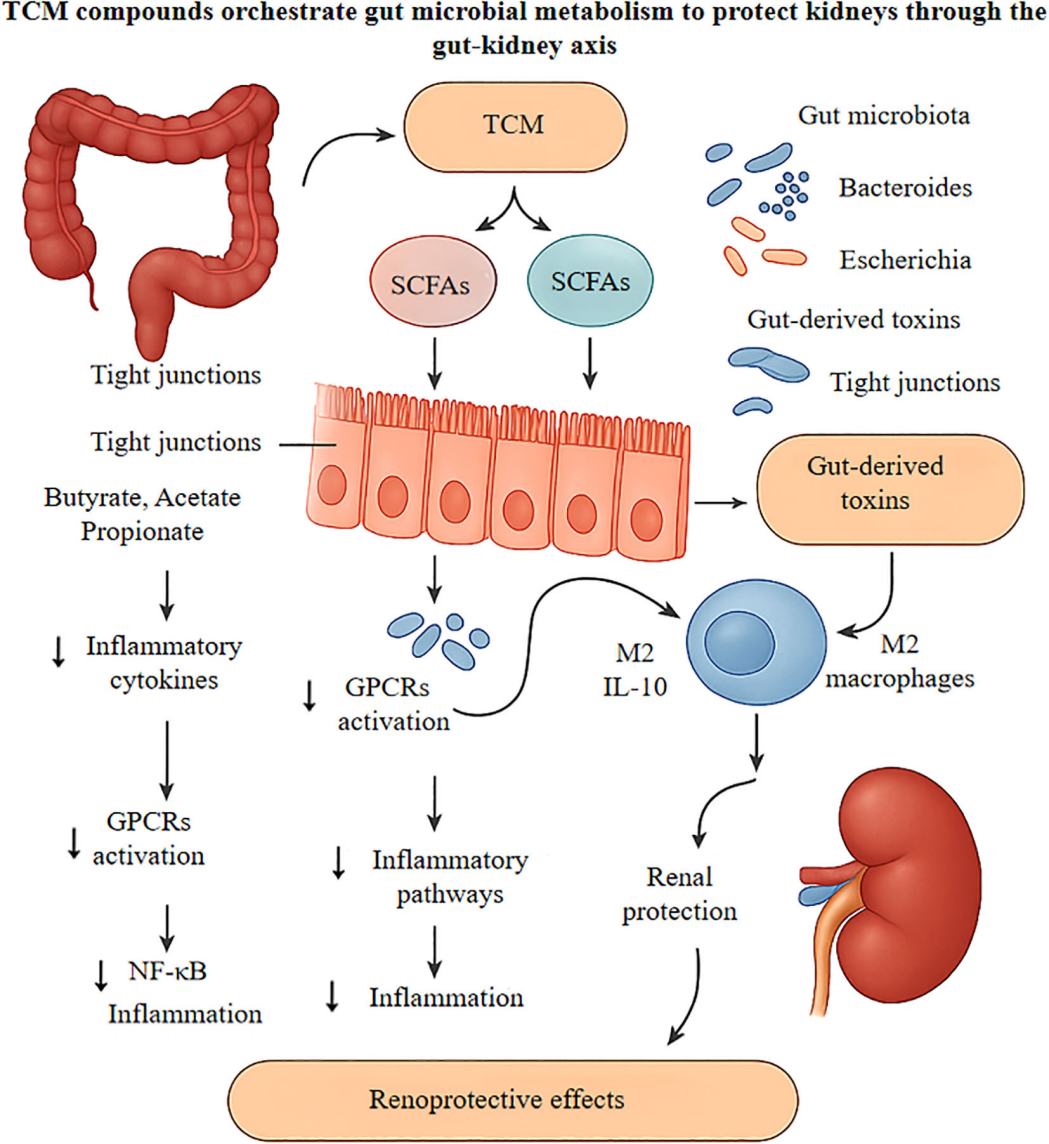
Delineates the nephroprotective mechanisms of traditional Chinese medicine compounds, particularly Astragalus polysaccharides and Rhizoma coptidis alkaloids, in ameliorating diabetic kidney disease progression via gut-kidney axis modulation. Oral administration of these bioactive compounds reshapes the intestinal microbiome by enriching beneficial commensals like Bacteroides and Lactobacillus while suppressing pathobionts including Escherichia coli, consequently diminishing gut-derived uremic toxins such as indoxyl sulfate. The enhanced microbial fermentation generates short-chain fatty acids, which upon entering circulation, engage G-protein coupled receptors to attenuate NF-κB-mediated renal inflammation and function as histone deacetylase inhibitors to epigenetically upregulate anti-inflammatory genes. Locally, these metabolites strengthen intestinal tight junctions, preventing endotoxin translocation. Additionally, SCFAs drive M2 macrophage polarization and suppress NLRP3 inflammasome activation, reducing IL-1β and IL-18 release while promoting IL-10 secretion. These orchestrated effects culminate in decreased glomerular podocyte injury and tubulointerstitial fibrosis, ultimately preserving renal function in diabetic nephropathy. TCM, Traditional Chinese Medicine; SCFAs, Short-Chain Fatty Acids; GPCRs, G-protein coupled receptors; NF-κB, Nuclear factor kappa-B; HDAC, Histone deacetylase; IL, Interleukin; NLRP3, NLR family pyrin domain containing 3.

This dual protection is further reinforced by SCFA-mediated activation of renal GPR43 and GPR109A receptors, which suppress inflammatory cascades within renal tissues (51). Meanwhile, reduced circulating toxin levels protect tubular epithelial cells from direct cytotoxicity (51, 59). Together, these microbiota-targeted interventions lead to improved renal outcomes, including decreased serum creatinine, attenuated glomerulosclerosis, and slowed fibrosis progression (105). This evidence supports the gut–kidney axis as a therapeutic target wherein TCM orchestrates microbiome modulation to exert systemic and renal protection (**Figure 3**).

## 4 Molecular mechanisms of the TCM–SCFA axis in delaying DKD-associated renal fibrosis

### 4.1 SCFA-mediated anti-inflammatory molecular mechanisms

SCFAs combat inflammation through distinct yet complementary molecular pathways that converge to suppress renal damage. First, SCFAs directly target inflammatory signaling by binding to GPR43, which triggers a cascade that dismantles the TLR4/MyD88 complex and stabilizes IκBα. This dual action blocks NF-κB from entering the nucleus and halts the production of pro-inflammatory cytokines including TNF-α, IL-6, and IL-1β (98, 99). Simultaneously, SCFAs function as potent HDAC inhibitors, promoting histone acetylation that drives IL-10 expression—a key anti-inflammatory mediator. This epigenetic reprogramming further dampens inflammation by preventing IKK phosphorylation, creating a reinforcing anti-inflammatory loop that protects renal tissue (106, 107).

Suppression of NLRP3 inflammasome activation represents a key mechanism by which the TCM–SCFA axis mitigates renal inflammation and fibrosis in DKD. SCFAs limit NLRP3 inflammasome assembly by reducing mitochondrial reactive oxygen species (mtROS) production and oxidative stress signaling, thereby preventing NLRP3 oligomerization and activation (107, 108). Additionally, SCFAs inhibit caspase-1 auto-activation and the cleavage of pro-IL-1β, which collectively lowers mature IL-1β secretion and dampens downstream inflammatory amplification (109, 110). These anti-inflammatory actions are further potentiated by TCM-derived phytochemicals, which synergistically suppress NLRP3/ASC/caspase-1 complex formation, reduce IL-1β maturation, and modulate TGF-β/Smad pathway phosphorylation, ultimately attenuating renal fibrotic responses (111, 112).

TCM potentiates the effects of SCFA regulation by promoting the proliferation of butyrate-producing bacteria via the microbiota–metabolite axis, thereby increasing SCFA concentrations (113, 114). These elevated SCFAs activate GPR43 receptors, which in turn suppress NLRP3 inflammasome activation and downregulate TGF-β-mediated Smad2/3 phosphorylation, leading to reduced collagen deposition (111, 112). In parallel, SCFA-dependent pathways modulate NF-κB signaling, thereby suppressing TGF-β-induced EMT and delaying tissue fibrosis progression (107, 112).

Evidence from DKD-specific models underscores the protective role of SCFAs and their associated pathways in mitigating core renal damage. *In vitro*, treating human kidney 2 (HK-2) cells with butyrate under high glucose conditions—mimicking the diabetic renal environment—significantly suppresses NLRP3 inflammasome activation and IL-1β release, thereby reducing tubular inflammation (51). Complementary *in vivo* findings in non-obese diabetic Akita mice reveal that acetate supplementation mitigates glomerular injury, evidenced by preservation of WT1-positive podocytes and reduced glomerular collagen IV deposition, indicating direct renoprotection at the structural level (51).

### 4.2 Blocking renal fibrosis: epigenetic and metabolic strategies

Preventing kidney fibrosis requires disrupting the TGF-β/Smad pathway through two converging mechanisms: epigenetic regulation and metabolic reprogramming (113, 114).

Butyrate, a key SCFA, acts as an HDAC inhibitor, increasing acetylation at histone residues H3K9 and H3K27 to open chromatin structure and suppress TGF-β1 transcription (115). This epigenetic remodeling reduces Smad2/3 phosphorylation and downstream deposition of collagen I/III and fibronectin. Additionally, butyrate inhibits EZH2-mediated H3K27 trimethylation, upregulating Smad7—a natural antagonist of TGF-β/Smad3 signaling that provides negative feedback inhibition (116, 117). TCM-derived phytochemicals amplify these effects. Curcumin prevents Smad7 degradation, maintaining its inhibitory action on TGF-β receptors and thus slowing glomerulosclerosis (118, 119). Beyond this, curcumin alleviates fibrosis by downregulating inflammatory cytokines and modulating key profibrotic signaling pathways in renal cells (120, 121). Puerarin operates via a different mechanism by upregulating miR-342-3p, which inhibits the TGF-β/Smad axis (122), and simultaneously attenuates fibrosis by suppressing oxidative stress and ferroptosis (123). Another compound, altenusin, blocks EMT in tubular epithelial cells, thereby reducing abnormal ECM accumulation and contributing to fibrosis prevention (124, 125). This direct antifibrotic effect involves antagonism of the TGF-β/Smad signaling pathway in renal proximal tubular cells (126). Mechanistic studies isolating the impact of hyperglycemia confirm the direct action of TCM-derived compounds on core DKD pathology, independent of obesity-related confounders. For example, investigations in experiments in streptozotocin (STZ)-induced diabetic rats demonstrate that curcumin directly inhibits TGF-β1–induced Smad2/3 phosphorylation within renal cortical tissues, thereby reducing ECM accumulation in both glomeruli and the tubulointerstitium (127). *In vitro* experiments using high glucose-stimulated mouse podocytes further reveal that berberine—a major active component of Huanglian Jiedu Decoction—ameliorates podocyte injury and cytoskeletal disruption by enhancing autophagy via activation of the AMPK/ULK1 pathway (128). These findings collectively highlight the ability of TCM compounds to directly target fundamental fibrotic processes within the kidney parenchyma, independent of systemic metabolic factors such as obesity​.

In addition to epigenetic regulation, metabolic reprogramming targets lipotoxicity in DKD. TCM-derived gypenosides activate the AMPK/PGC-1α signaling pathway, promoting a metabolic shift in renal tubular epithelial cells from fatty acid synthesis toward fatty acid oxidation (129–131). This reprogramming alleviates intracellular lipid accumulation and reduces the expression of fibrotic markers, including α-smooth muscle actin (α-SMA) and plasminogen activator inhibitor-1 (PAI-1). Glycolysis exerts dual effects: while excessive glycolytic flux can induce histone lactylation, particularly H4K12la, thereby driving proinflammatory gene expression, inhibition of the glycolytic enzyme PFKFB3 has been shown to prevent this modification and attenuate oxidative injury (132, 133). Butyrate coordinates these metabolic pathways by upregulating miR-29b, which simultaneously suppresses collagen I and III gene expression and enhances glycolysis to sustain energy production under cellular stress (134). Additionally, miR-29b downregulates SETD2, thereby modulating H3K36me3 levels and further inhibiting TGF-β signaling (117, 134).

**Figure 4:**
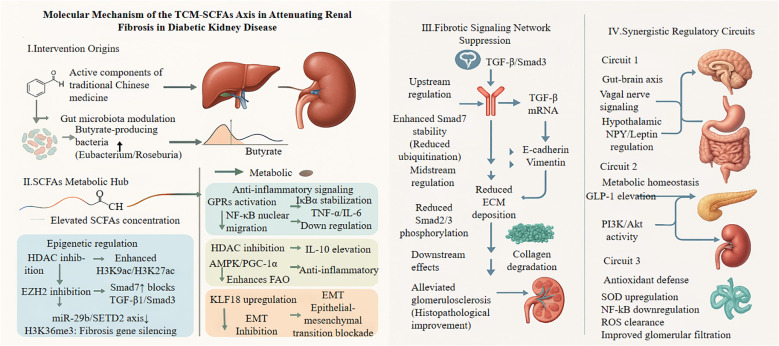
This schematic depicts how Traditional Chinese Medicine (TCM) components modulate gut microbiota to suppress renal fibrosis through SCFA-mediated pathways. TCM-derived compounds (astragalus polysaccharides, resveratrol, berberine) promote butyrate-producing bacteria (Eubacterium, Roseburia), increasing circulating SCFAs. These metabolites act as HDAC inhibitors, enhancing histone acetylation and Smad7 expression while suppressing the miR-29b/SETD2 axis. In renal cells, SCFAs activate GPR43/41 receptors, triggering AMPK and inhibiting NF-κB. This cascade blocks TGF-β/Smad3 signaling at multiple levels: Smad7 prevents receptor phosphorylation, Smad2/3 nuclear translocation is reduced, and profibrotic gene expression (EMT markers, ECM components) is suppressed. Three synergistic circuits amplify renoprotection: (1) gut-brain axis modulation via vagal signaling; (2) metabolic homeostasis through GLP-1 secretion, activating renal PI3K/Akt pathways; (3) enhanced antioxidant defense (SOD upregulation) combating oxidative stress. Together, these mechanisms preserve glomerular function and reduce albuminuria in diabetic kidney disease. GPRs, G-protein coupled receptor; IκBα, Inhibitor of κ B α; AMPK, Adenosine monophosphate-activated protein kinase; PGC-1α, Peroxisome proliferator-activated receptor γ coactivator 1-α; EZH2, Enhancer of zeste homolog 2; KLF18, Kruppel-like factor 18; EMT, Epithelial-mesenchymal transition; SETD2, SET domain-containing 2; Smad3, Mothers against dpp homolog 3; GLP-1, Glucagon-like peptide-1; PI3K, Phosphatidylinositol 3-kinase; Akt, Protein kinase.

Synergistic mechanisms amplify protection against renal fibrosis through coordinated epigenetic and metabolic regulation. Epigenetic enzymes such as HDACs and EZH2 regulate both fibrotic gene expression and metabolic enzyme activity, forming reinforcing feedback loops that sustain pathological signaling (51, 115, 117, 135). TCM formulations strategically exploit these interactions. For instance, Huanglian Jiedu Decoction promotes miR-663a expression to inhibit the lncRNA C18orf26-1/TGF-β/Smad axis, effectively blocking EMT and ECM deposition (118, 119, 136, 137). Liposomal gypenosides activate AMPK and suppress Smad2/3 phosphorylation, targeting fibrosis through both metabolic and epigenetic pathways (129–131). A comprehensive overview of the molecular mechanisms by which the TCM–gut microbiota–SCFAs axis attenuates renal fibrosis is illustrated in **Figure 4**. This conceptual model delineates a stepwise sequence: (I) TCM-derived phytochemicals selectively enrich butyrate-producing gut bacteria; (II) Elevated SCFA levels act as signaling molecules and potent epigenetic modifiers—functioning as HDACi and EZH2i inhibitors—to suppress profibrotic gene expression and stabilize inhibitory regulators such as Smad7; (III) SCFAs directly inhibit renal fibrotic signaling, particularly the TGF-β/Smad3 pathway, by activating GPR that engage AMPK and NF-κB signaling, increasing Smad7 expression, inhibiting Smad2/3 phosphorylation, and preventing EMT blockade; (IV) Synergistic effects are further reinforced through gut–brain axis crosstalk, glucagon-like peptide-1 (GLP-1)-mediated metabolic improvements, and enhanced antioxidant defenses within renal tissue. The convergence of these direct and indirect mechanisms results in the attenuation of ECM accumulation and preservation of renal structure, offering a multi-layered strategy for delaying the progression of DKD.

### 4.3 Other synergistic mechanisms

#### 4.3.1 Improving metabolic homeostasis

SCFAs, particularly acetate and propionate—major end products of intestinal microbial fermentation—exert beneficial systemic effects by activating GPR41 and GPR43 receptors on enteroendocrine L cells. This stimulation promotes the secretion of GLP-1, a key incretin hormone that enhances insulin secretion from pancreatic β-cells and modulates hepatic glucose and lipid metabolism (138). GLP-1 improves insulin sensitivity, facilitates glucose uptake, and regulates lipid metabolism, thereby alleviating glycolipid metabolic disturbances commonly associated with hyperglycemia and hyperlipidemia in DKD (139, 140). These actions involve the activation of GLP-1–dependent PI3K/Akt signaling, which promotes glucose transport and fatty acid oxidation while reducing hepatic glycogenolysis and lipid accumulation (121, 123, 139, 141). Beyond metabolic effects, SCFAs also confer antioxidant and anti-inflammatory benefits. They stimulate the expression of antioxidant enzymes such as superoxide dismutase and inhibit NF-κB–driven pro-inflammatory pathways, thereby counteracting hyperglycemia-induced glomerular hyperfiltration and oxidative stress (142, 143). This leads to reduced ROS production and preserves the structural and functional integrity of renal cells (144, 145). Collectively, these mechanisms demonstrate how SCFAs contribute to restoring metabolic homeostasis by balancing energy metabolism and suppressing inflammation, ultimately mitigating renal damage in DKD (128, 129, 146, 147).

TCM components, including ginseng and astragalus, modulate the composition of the intestinal microbiota by increasing the abundance of beneficial bacteria, thereby enhancing SCFA production, particularly butyrate and acetate (122, 130, 140, 148). These SCFAs activate GPR41 receptors, initiating gut–brain axis signaling that influences hypothalamic regulation of appetite-controlling neuropeptides such as neuropeptide Y and leptin (149–151). Through vagal afferent pathways, this signaling suppresses hypothalamic appetite centers, thereby reducing food intake and lipid accumulation (149, 150). Indirectly, this mechanism ameliorates obesity-related DKD by lowering systemic levels of inflammatory mediators such as TNF-α and IL-6, improving insulin sensitivity, and ultimately inhibiting glomerulosclerosis and fibrosis progression (138, 145). Supporting evidence highlights the critical role of gut–brain axis dysregulation in glycolipid metabolic disorders, and the restoration of microbial balance via TCM therapy has been shown to improve metabolic phenotypes (149–151). In addition to these indirect mechanisms, multiple TCM formulations also exert direct anti-fibrotic effects. For example, Huangqi decoction and Jiedu Tongluo Baoshen (JTBF) formulations have been shown to attenuate renal fibrosis by targeting key profibrotic pathways such as TGF-β/MAPK (152, 153). Similarly, Sang-Bai-Pi extract alleviates renal fibrosis by simultaneously inhibiting the TGF-β/Smad and Wnt/β-catenin cascades, illustrating the capacity of TCM-derived compounds to directly disrupt fibrotic signaling networks (13, 124). Furthermore, other natural compounds, including fibroblast growth factor 21 (FGF21), have demonstrated anti-fibrotic efficacy through downregulation of the Wnt/β-catenin pathway (154). These findings emphasize the multifaceted roles of TCM interventions in modulating metabolic, inflammatory, and fibrotic pathways central to DKD pathogenesis.

#### 4.3.2 Evidence from preclinical models

Preclinical studies using diabetic animal models, including db/db mice, have demonstrated that TCM interventions—particularly polyphenol extracts—can significantly attenuate renal injury, as evidenced by reduced urinary protein excretion and improved glomerular morphology (140, 148). These renoprotective effects are strongly associated with gut microbiota remodeling, characterized by increased abundance of beneficial taxa such as Verrucomicrobia and increased production of SCFA, particularly butyrate and acetate (140, 148, 155). SCFAs enhance intestinal barrier integrity and restore immune homeostasis by reducing Th17/Treg ratios, thereby mitigating glomerular inflammation and oxidative stress (143, 145, 151). Moreover, increased SCFA levels are positively correlated with improved insulin sensitivity, which helps reduce the hyperglycemia-induced filtration burden on the kidneys (139, 140, 155). Among these TCM compounds, resveratrol has been particularly well-studied. In obese mouse models, resveratrol treatment reshapes gut microbiota composition by suppressing pathogenic bacteria and promoting butyrate-producing species (145). The resulting elevation in butyrate levels exerts direct antifibrotic effects in renal tissue by inhibiting phosphorylation of the TGF-β/Smad3 signaling axis, thereby reducing fibroblast activation and type IV collagen deposition (145, 156). In parallel, the microbiota–SCFA axis alleviates oxidative stress and inflammation by promoting ROS clearance and downregulating TGF-β expression (142, 145). Additionally, resveratrol-induced microbiota modulation has been shown to improve gut–brain axis signaling, contributing to broader metabolic protection (149, 151, 155). Collectively, these findings provide robust preclinical evidence that TCM-induced microbiota remodeling and SCFA elevation represent a key therapeutic mechanism for attenuating DKD.

## 5 Clinical-associated studies

Although most research on the interplay between TCM, gut microbiota, and DN remains at the preclinical stage, emerging clinical evidence validates the therapeutic potential of this approach (157).

Patients with CKD and DN consistently exhibit gut dysbiosis. A cross-sectional study spanning various CKD stages revealed that declining renal function associates with a marked reduction in butyrate-producing bacteria and a concomitant increase in opportunistic pathogens, underscoring the pathophysiological rationale for microbiota-targeted therapies (50).

Clinical studies have also begun to demonstrate the efficacy of TCM-based interventions. Several TCM formulations have demonstrated promising clinical efficacy. In a multicenter randomized controlled trial (RCT, n = 88), administration of Huangkui capsule (derived from Abelmoschus manihot) alongside standard therapy significantly reduced urinary albumin excretion and attenuated eGFR decline compared to placebo over an 8-week period (ChiCTR-OON-17012076) (158). Notably, this treatment also improved gut microbiota composition, supporting a gut–kidney axis mechanism (159). Similarly, another RCT involving 118 patients with DKD demonstrated that adjunctive APS therapy led to significant reductions in 24-hour proteinuria and serum creatinine, while concurrently enhancing gut microbial diversity (NCT03535935) (160). These findings provide early clinical validation of TCM-based gut microbiota modulation as a viable strategy for mitigating DN progression.

Emerging clinical evidence underscores the relationship between circulating SCFAs and kidney function preservation in DKD. A cohort analysis involving 100 patients demonstrated that elevated serum SCFA levels were significantly associated with better maintenance of eGFR (P < 0.05), implicating SCFA deficiency as a potential contributor to progressive renal deterioration (28). A registered trial (NCT04459156) employing tracer pulse methodology demonstrated increased SCFA production following prebiotic interventions, suggesting potential renoprotective benefits (50). Population-based studies, including those derived from the NHANES database, have similarly linked specific fatty acid profiles with renal function decline in individuals with type 2 diabetes mellitus (T2DM), reinforcing the clinical relevance of SCFA modulation (161, 162). In parallel, microbiota-targeted strategies beyond TCM are gaining attention. Ongoing clinical trials evaluating probiotic supplementation in patients with early-stage DN report initial findings of reduced uremic toxin levels and elevated circulating butyrate concentrations. Complementary observational studies in patients with DKD receiving herbal formulations have documented improvements in intestinal barrier function and reductions in circulating endotoxin levels, findings that align with preclinical models and further support the therapeutic potential of targeting the microbiota–gut–kidney axis in DKD (163).

Despite encouraging preliminary results, the clinical application of TCM for DKD via modulation of SCFAs remains limited by several research challenges. Large-scale, multicenter RCTs directly validating the renoprotective effects of TCM-mediated SCFA regulation in DKD are notably lacking. Future studies should adopt integrated multi-omics strategies—such as metagenomics and metabolomics—to dynamically monitor gut microbiota and SCFA profiles, correlating these with clinical outcomes to provide high-quality evidence for the therapeutic utility of TCM in DKD management (164).

## 6 Research limitations, clinical prospects, and future directions

### 6.1 Research limitations

Although growing evidence supports the potential of TCM to modulate the gut microbiota–SCFA axis in DKD, several critical limitations hinder clinical translation. First, the underlying molecular mechanisms remain incompletely understood. While TCM-derived polysaccharides and polyphenols have been shown to elevate SCFA levels—particularly butyrate and propionate—through gut microbiota modulation and ameliorate renal fibrosis (47), the specific microbial enzymatic pathways responsible for SCFA biosynthesis remain undefined, despite evidence that TCM polysaccharides such as APS promote probiotic growth (162, 164). Moreover, the interactions between SCFAs and host receptors, especially GPR41 and GPR43, lack comprehensive validation in the context of DKD (5). Downstream mechanisms, such as NF-κB signaling inhibition, remain speculative without direct molecular evidence (15). Mechanistic studies are also often limited by their reliance on comorbidity models involving diabetes and obesity (165), rather than isolating core DKD pathologies such as glomerular and tubular injury. Furthermore, high-quality human data are scarce; most findings are derived from preclinical animal models or *in vitro* models (88), with a notable absence of large-scale RCTs evaluating the efficacy of TCM in modulation of the microbiota–SCFA axis in patients with DKD (52, 166). Finally, current studies lack a dynamic, longitudinal monitoring of SCFA levels in fecal or blood samples, making it difficult to establish clear associations between SCFA fluctuations and renal outcomes (26).

#### 6.1.1 Insufficient attention to microbial community functional heterogeneity

Individual baseline gut microbiota composition may significantly influence TCM therapeutic efficacy (159, 167). However, most existing clinical trials do not stratify participants based on their initial microbiota characteristics—such as Firmicutes/Bacteroidetes ratios or the abundance of SCFA-producing bacteria—leaving the heterogeneity in treatment responses largely unexplained. Dysbiosis is common in DKD, including reduced levels of Lactobacillus and may influence how patients respond to TCM interventions, yet this remains poorly investigated (165). Furthermore, environmental confounders such as diet, antibiotic use, and other lifestyle factors are often overlooked in efficacy assessments, potentially obscuring genuine treatment effects (159).

#### 6.1.2 Other critical limitations

The therapeutic potential of SCFAs as clinical targets remains underexplored. Although compounds such as butyrate demonstrate renoprotective effects, their effective concentration thresholds and long-term safety as clinical biomarkers or therapeutic agents have not been systematically assessed (165, 168). Furthermore, most studies lack integrated multi-omics approaches—comprehensive analyses combining microbiome profiles (microbial community structure), metabolome data (SCFA concentrations), and host transcriptome or epigenome alterations—thus limiting construction of a holistic mechanistic framework (50). Microbiota transplantation also faces significant translational challenges, as no consensus exists regarding the optimal composition of functional microbial communities or standardized transplantation protocols specifically tailored for DKD, thereby limiting clinical applicability (28, 50, 164).

### 6.2 Clinical prospects and future directions

In the clinical management of DKD, future research should prioritize the elucidation of underlying mechanisms, with multi-omics technologies serving as pivotal tools. Metagenomic analyses, such as 16S rDNA sequencing, can characterize gut microbiota dysbiosis in DKD, particularly identifying reductions in SCFA-producing taxa like *Clostridium butyricum* (161, 169). Complementary metabolomic approaches, including gas chromatography-mass spectrometry, allow for precise quantification of key SCFAs such as butyrate and propionate, thereby advancing our understanding of metabolite fluctuations in relation to disease progression (161). Preclinical evidence demonstrates that SCFAs, particularly butyrate, exert renoprotective effects by suppressing pro-inflammatory cytokines like IL-6 and TNF-α, underscoring their immunomodulatory potential in DKD (169, 170). However, these findings are largely based on murine models, necessitating validation in human populations with larger, more diverse sample sizes (171).

Gut dysbiosis has been strongly linked to DKD pathogenesis, characterized by reduced microbial diversity and decreased abundance of SCFA-producing bacteria. These microbial alterations correlate significantly with markers of renal dysfunction, including eGFR decline and elevated urine albumin-to-creatinine ratio (UACR) (39, 172), supporting the rationale for stratified patient management (163). Based on this foundation, future phase III RCTs could adopt microbiota-guided stratification strategies. For instance, individuals with specific dysbiotic microbiota profiles could be preferentially assigned to receive Huangkui capsule, a TCM shown to modulate microbial composition and mitigate renal pathological injury in DKD (173). This design aims to validate the differential therapeutic efficacy of targeted interventions in patients with specific microbial phenotypes. By screening microbiota-responsive populations and leveraging Huangkui capsule’s regulatory effects on pathogenic microbiota (173), this approach facilitates the development of precision treatment models linking DKD-associated microbiota subtypes with TCM therapeutic responses (173, 174). Such methodology holds promise for advancing clinical translation and optimizing individualized therapeutic strategies.

Regarding clinical research optimization, robust associations between gut microbiota characteristics and DKD progression have been established. Specifically, microbiota compositional changes, such as reduced diversity and lower abundance of SCFA-producing bacteria, are significantly correlated with renal function indicators—notably declining eGFR and elevated UACR—providing a rationale for individualized patient stratification (172, 175). Moreover, findings from animal models demonstrate that fluctuations in SCFA levels often parallel renal function impairment, supporting the feasibility of using SCFAs—particularly butyrate—as biomarkers in blood or feces (161, 176). However, SCFA detection methodologies require standardization to ensure reproducibility and clinical reliability (167, 171).

**Figure 5:**
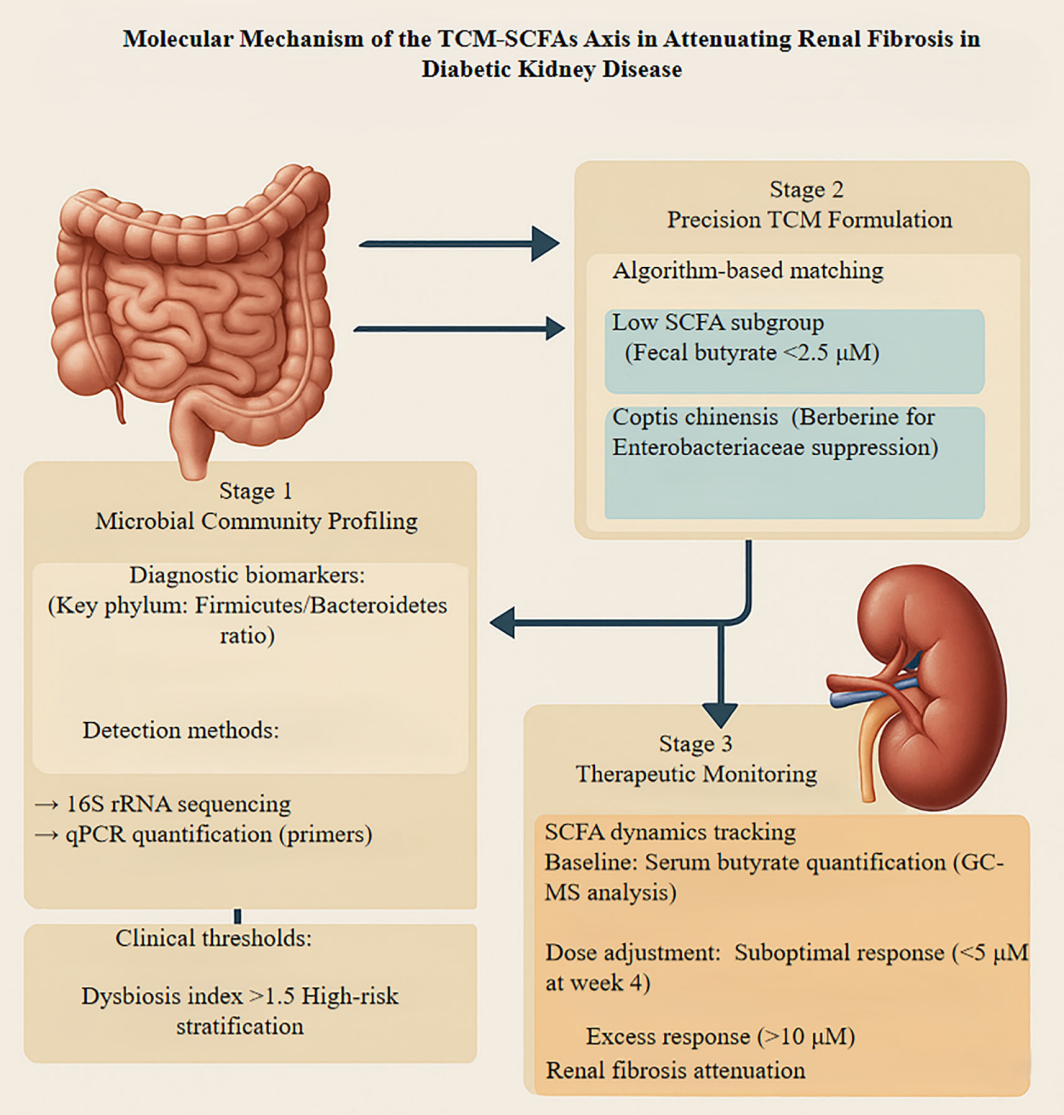
Presents a translational framework integrating traditional Chinese medicine with microbiome modulation to mitigate diabetic kidney disease-associated renal fibrosis through precision therapeutics. The approach encompasses three iterative phases: initial microbial dysbiosis profiling employing 16S rRNA sequencing and qPCR quantification to identify patients with elevated Firmicutes/Bacteroidetes ratios (>1.5) and diminished butyrate-producing capacity. Subsequently, an algorithm-guided intervention stratifies patients with fecal butyrate levels below 2.5 μM for targeted TCM formulations, incorporating berberine-rich Coptis chinensis to suppress pathogenic Enterobacteriaceae while enhancing beneficial commensals. The final phase involves dynamic monitoring of serum butyrate concentrations via GC-MS, with protocol adjustments implemented when levels deviate from the therapeutic window (5-10 μM). Renal outcomes are validated through histological assessment of collagen deposition and TGF-β expression. This iterative feedback system enables personalized dosing optimization, representing a paradigm shift toward precision nephrology that bridges traditional pharmacotherapy with contemporary microbiome science. TCM, Traditional Chinese Medicine; SCFA, Short-Chain Fatty Acid; rRNA, Ribosomal RNA; 16S, 16S ribosomal RNA; qPCR, Quantitative Polymerase Chain Reaction; GC-MS, Gas Chromatography-Mass Spectrometry.

For microbiota-targeted therapy, microbial intervention strategies include the use of specific probiotic strains that enhance SCFA production, as well as prebiotics such as dietary fiber, which increase SCFA production and mitigate renal injury (9, 43, 58). Additionally, natural compounds like resveratrol modulate gut microbiota composition, resulting in elevated SCFA levels and subsequent attenuation of renal fibrosis (39). In support of causal mechanisms, Mendelian randomization analyses have provided genetic evidence for associations between the gut microbiota and DKD. Specifically, changes in the abundance of SCFA-producing bacterial genera have demonstrated causal relationships with renal injury risk, where reductions in key microbial populations are associated with negative renal outcomes (158, 177). To strengthen causal inference, future studies should integrate clinical datasets, combining metagenomic profiles with renal function markers such as eGFR, while carefully controlling for confounding factors (158, 176). Overall, future research should prioritize mechanistic elucidation, clinical translation, and optimization of therapeutic strategies to facilitate the development of precision medicine approaches in DKD. **Figure 5** outlines a proposed roadmap for this clinical translation.

## 7 Discussion

This review has systematically elucidated the mechanistic framework by which TCM ameliorates DN-associated renal fibrosis through modulation of the gut microbiota–SCFA axis. Our analysis demonstrates that TCM exerts therapeutic efficacy through a multi-tiered regulatory network that involves both microbiota-mediated and renal cell-targeted mechanisms.

TCM interventions improve DN outcomes primarily via three interrelated pathways. First, TCM components promote intestinal microbial homeostasis by increasing the abundance of SCFA-producing bacteria (particularly Fecalibacterium and Lactobacillus) while suppressing pathogenic taxa. This microbial remodeling significantly enhances SCFA biosynthesis—particularly butyrate and acetate—which act as key metabolic mediators along the gut–kidney axis. Second, elevated SCFAs, especially butyrate, exert anti-inflammatory effects by inhibiting NF-κB signaling, thereby reducing pro-inflammatory cytokine expression and macrophage infiltration in renal tissue. Third, SCFA-driven signaling downregulates fibrotic pathways, notably by suppressing TGF-β/Smad activation and reducing ECM accumulation, ultimately mitigating renal fibrosis progression.

Notably, emerging clinical evidence, including RCTs of TCM formulations and observational studies linking SCFA levels with renal function, supports the translational potential of this therapeutic strategy. The identification of predictive biomarkers, such as butyrate concentrations and Bacteroidetes abundance, marks a significant step toward realizing personalized medicine in DN management. Several key research priorities must be addressed to strengthen mechanistic understanding and optimize therapeutic application. Mechanistic studies should focus on elucidating the SCFA–GPR signaling networks and their epigenetic regulatory roles in renal cells. Clinically, large-scale multicenter trials incorporating multi-omics approaches are essential to establish robust evidence for TCM efficacy and guide the development of standardized treatment protocols. Additionally, the formulation of novel TCM therapies specifically designed to enhance SCFA production while reducing uremic toxin generation presents a promising direction for therapeutic innovation. Despite these promising findings, some limitations should be acknowledged. First, the compositional variability and bioavailability of active compounds in TCM formulations introduce challenges in standardization, dosage consistency, and reproducibility across studies. Second, the causal relationships between specific microbial taxa, SCFA levels, and renal outcomes remain insufficiently characterized, especially in the presence of confounding factors such as diet, antibiotic exposure, and disease heterogeneity.

In conclusion, the gut microbiota–SCFA axis represents a pivotal and modifiable target in DN management, with TCM offering a distinctive and effective approach to modulate this axis. By integrating traditional pharmacology with contemporary biomedical science, TCM-based strategies hold considerable promise for improving outcomes in DN and may reshape the therapeutic landscape of this complex diabetic complication.
